# 

**Published:** 1906-04-07

**Authors:** 


CONTENTS.

Daily Practice and Clinical Expert Teaching - - 1
Annotations - - - - - - - - - - - - - 2
The Facial Aspect in Diseases of Children - - 3
Spirochæta Pallida of Schaudiun - - - - - - - 5
Psoriasis - - - - - - - - - - - - - - 5
Spastic Constipation - - - - - - - - - - 6
The Book World of Medicine and Science - - - 7
The Hospital Special Analytical Commission on Whisky - - - - - - - - 8
New Appliances and Things Medical - - - - 19
Medical Appointments and Vacancies - - - - 19
Hospital Administration—
The Growth of Hospital Expenditure considered in Relation to Greater London - - - - - - - - - 20
Speyer Prize Competition - - - - - - - 23
League of Mercy - - - - - - - - - 23
Hospital Meetings - - - - - - - - - 24
Practical Departments - - - - - - - 25
Editor's Letter-Box - - - - - - - - 26
Notes and News - - - - - - - - - - 26

*NURSING SECTION.*

Notes on News from the Nursing World - - - 1-4

The Nursing Outlook - - - - - - - - 5

The Care and Nursing of the Insane - - - - 6

The Nurses' Clinic - - - - - - - - 7

Incidents in a Nurse's Life - - - - - - 8

Nursing at Sheffield in the Eighties - - - - 9

On Practical Nursing - - - - - - - - 11

Camps for Consumptives - - - - - - - 13

What Nurses Owe to the Medical Profession - - 14

A Medical Mission Hospital in India - - - - 15

The Training of Cottage Nurses - - - - - 17

Central Midwives Board - - - - - - - - 18

A Nightingale Nurse on the Pension Fund - - 19

Poor Law and the Central Midwives Board - - 19

Practical Hints - - - - - - - - - - 20

Everybody's Opinion - - - - - - - - 20

Appointments - - - - - - - - - - - 22

Novelties for Nurses - - - - - - - - 22

A Book and Its Story - - - - - - - - 24

Death in Our Ranks - - - - - - - - 25

Notes and Queries - - - - - - - - - 25

For Reading to the Sick - - - - - - - 26

